# FTY720 Attenuates LPS-Induced Inflammatory Bone Loss by Inhibiting Osteoclastogenesis via the NF-*κ*B and HDAC4/ATF Pathways

**DOI:** 10.1155/2023/8571649

**Published:** 2023-01-06

**Authors:** Chongwei Chen, Sujing Zong, Zhenyu Wang, Ruijia Yang, Yanjing Guo, Yunfei Wang, Xinping Chen, Yue Li, Shaowei Wang

**Affiliations:** ^1^Shanxi Key Lab of Bone and Soft Tissue Injury Repair, Department of Orthopaedics, The Second Hospital of Shanxi Medical University, Taiyuan, China; ^2^Department of Biochemistry, Basic Medical College, Shanxi Medical University, Taiyuan, China; ^3^Shanxi Bethune Hospital, Shanxi Academy of Medical Sciences, Tongji Shanxi Hospital, Third Hospital of Shanxi Medical University, Taiyuan, China

## Abstract

Osteoclast (OC) abnormalities lead to many osteolytic diseases, such as osteoporosis, inflammatory bone erosion, and tumor-induced osteolysis. Exploring effective strategies to remediate OCs dysregulation is essential. FTY720, also known as fingolimod, has been approved for the treatment of multiple sclerosis and has anti-inflammatory and immunosuppressive effects. Here, we found that FTY720 inhibited osteoclastogenesis and OC function by inhibiting nuclear factor kappa-B (NF-*κ*B) signaling. Interestingly, we also found that FTY720 inhibited osteoclastogenesis by upregulating histone deacetylase 4 (HDAC4) expression levels and downregulating activating transcription factor 4 (ATF4) expression levels. In vivo, FTY720 treatment prevented lipopolysaccharide- (LPS-) induced calvarial osteolysis and significantly reduced the number of tartrate-resistant acid phosphatase- (TRAP-) positive OCs. Taken together, these results demonstrate that FTY720 can inhibit osteoclastogenesis and ameliorate inflammation-induced bone loss. Which may provide evidence of a new therapeutic target for skeletal diseases caused by OC abnormalities.

## 1. Introduction

In normal bone tissue, homeostasis is maintained by the delicate balance between osteoblast-mediated bone formation and osteoclast- (OC-) mediated bone resorption, and dysregulation of this balance can lead to a variety of skeletal disorders [1, 2]. Excessive OC activation and OC dysfunction disrupt the balance between bone formation and bone resorption, thus leads to increased bone resorption and results in most skeletal diseases, such as pathological bone loss or reduction, osteoporosis, and Paget's disease [3, 4]. However, there are no effective treatments for OC-related skeletal diseases. Therefore, it is essential to explore effective inhibitors of OC-mediated bone resorption.

OCs, a class of cells that differ from monocyte- or macrophage-lineage cells, can resorb bone [5]. OC formation and function require two crucial cytokines: macrophage colony-stimulating factor (M-CSF) and receptor activator of NF-*κ*B ligand (RANKL) [6]. OCs express receptor activator of nuclear factor kB (RANK), and the binding of RANK with its ligand, RANKL, initiates the recruitment of tumor necrosis factor receptor-related factor 6 (TRAF6), thereby activating signaling pathways such as the mitogen-activated protein kinase (MAPK) and nuclear factor kappa-B (NF-*κ*B) signaling pathways, which supports RANKL-induced osteoclastogenesis by upregulating nuclear factor of activated T cells c1 (NFATc1) expression levels [7, 8]. Activation of NFATc1 by NF-*κ*B initiates the expression of OC-related genes that regulate osteoclastogenesis and differentiation [9]. Furthermore, osteoclastogenesis was found to be decreased after conditional knockout of NFATc1 [10]. The formation of OCs is a complex regulatory process, and the study of the mechanisms underlying the diseases caused by OC abnormalities may provide new therapeutic targets for the treatment of inflammatory bone loss.

Histone deacetylase 4 (HDAC4) is a member of the classic class IIa histone deacetylases (HDACs) (including HDAC4, HDAC5, HDAC7, and HDAC9) and participates in regulating cell growth and differentiation by altering chromosome structure and inhibiting the activity of specific transcription factors [11]. Class IIa HDACs are negative regulators of osteoclastogenesis [12–14]. In addition, HDAC4 plays a role in regulating endoplasmic reticulum stress-induced apoptosis by binding to and inhibiting the activity of activating transcription factor 4 (ATF4) [15, 16], which is a stress-induced transcription factor that plays an important role in cancer cell survival [17]. The research shows that OC differentiation is significantly reduced in bone marrow mononuclear cell cultures derived from ATF4 knockout mice [18]. Those indicate that OC differentiation depends on ATF4, and HDAC4 will be able to restrain osteoclastogenesis by inhibiting ATF4. It is very important to find out the mechanism above for studying the effect of drugs in osteoporosis and bone-related diseases, such as vitamin C and parathyroid hormone-related protein (PTHrP) [19, 20].

FTY720 (fingolimod) was approved by the United States Food and Drug Administration (FDA) in 2010 as the first immunomodulatory drug for the oral treatment of multiple sclerosis [21]. It acts as a sphingosine-1-phosphate (S1P) receptor modulator with neuroprotective effects, affecting myelin regeneration in multiple sclerosis [22], and is also used in ischemic stroke treatment [23]. FTY720 may act as an immunosuppressant for organ transplant rejection and cancer. In clinical trials to study transplanted organ rejection, treatment with FTY720 attenuated ovariectomy-induced osteoporosis, which demonstrates its effects on bone loss [24, 25]. FTY720, which also modulates multiple S1P receptors, can inhibit the release of proinflammatory cytokines and bone loss [26, 27]. In this work, we investigated how FTY720 affects osteoclastogenesis and OC function in vitro and LPS-induced bone loss in vivo.

## 2. Materials and Methods

### 2.1. Primary Cell Culture

Primary bone marrow cells were extracted from the femurs and tibias of 6-week-old male C57BL/6 mice and cultured in *α*-modified minimal essential medium (*α*-MEM, Gibco, USA) containing 10% fetal bovine serum (FBS, Gibco) and 1% PS (100 U/ml penicillin, 100 *μ*g/ml streptomycin, Thermo Fisher Scientific, USA), and 25 ng/ml M-CSF was added to induce bone marrow-derived macrophage (BMMs) formation [3, 4, 28]. The cells were cultured at 37°C in a cell incubator with 5% CO_2_ until they reached 90% confluence. Subsequently, the cells that did not adhere to the plate were removed, and the adherent cells were used for the next experiments.

### 2.2. Cell Activity Assay

The cytotoxic effect of FTY720 (MCE, China) on BMMs was studied using the cell counting kit-8 (CCK-8) assay (MCE, USA) according to the manufacturer's protocols. Briefly, BMMs were seeded in 96-well plates at a density of 3 × 10^3^ cells/well in *α*-MEM containing 25 ng/ml M-CSF (LifeTein, LLC, Beijing, China). After 24 h of incubation, the cells were administered FTY720 (0, 4, 8, 16, 32, 64, 128, 256, and 512 nM) for 48 h, 72 h, and 96 h. At the specified time point, 10 *μ*L of CCK-8 reagent was added to each well. The plate was incubated at 37°C for another 2 hours in the dark. An enzyme labeling instrument (Thermo Fisher Scientific, USA) was used to determine the absorbance at 450 nm.

### 2.3. Osteoclastogenesis and TRAP Staining Assays

BMMs were seeded into 96-well plates and cultured overnight in *α*-MEM containing M-CSF (25 ng/mL) to adhere, after which RANKL (50 ng/mL, LifeTein, LLC, Beijing, China) was added to the medium to induce the formation of OCs [29], which were then treated with different concentrations (0–512 nM) of FTY720. The culture medium was replaced every 48 h for 7 days. After OCs were observed in the control group, the cells were fixed, and TRAP staining was then performed using a TRAP staining kit (Sigma, USA) according to the manufacturer's instructions. In detail, the cells were fixed for 30 s and then stained with naphthol AS-BI and tartrate solution at 37°C for 1 h in the dark [30, 31]. The TRAP-positive multinucleated cells (with three or more nuclei) observed under a microscope were considered OCs.

### 2.4. RT–qPCR

BMMs were seeded in six-well plates at 1.5 × 10^5^ cells/well and cultured in *α*-MEM containing M-CSF (25 ng/mL) and RANKL (50 ng/mL) in the presence or absence of FTY720 (16 nM) for 5 days. We extracted total RNA from the cells using TRIzol reagent, and cDNA was then reverse transcribed with PrimeScript RT Master Mix (Takara, Beijing, China) according to the manufacturer's protocol. qPCR was performed using a TB Green Premix Ex Taq (Takara, Beijing, China) kit. The relative expression of target genes was normalized to the expression level of the housekeeping gene 18S based on the 2^−ΔΔCT^ method. The sequences of primers specific for each target gene (tartrate-resistant acid phosphatase 5 (Acp5), recombinant human cathepsin K (CTSK), vacuolar ATPase d2 (V-ATPase d2), dendritic cell-specific transmembrane protein (DC-STAMP), c-Fos, NFATc1, tumor necrosis factor receptor superfamily member 11a (TNFRSF11a), and 18S) are shown in Table 1. The primers were purchased from Sangon Biotech (Shanghai, China).

### 2.5. Western Blotting

BMMs were seeded in six-well plates at 1.5 × 10^5^ cells/well and cultured overnight for adherence. The cells were treated with medium containing M-CSF (25 ng/mL) and RANKL (50 ng/mL) with or without FTY720 (16 nM). Then, proteins were extracted from the cells with or without FTY720 stimulation for 1, 3, and 5 days using a whole-protein extraction kit. The above procedure used to obtain proteins was used for assays of c-Fos, NTATc1, integrin B3, HDAC4, ATF4, and myocyte enhancer factor 2C (MEF2C) protein expression.

To investigate the role of FTY720 in the RANKL-induced NF-*κ*B signaling pathway, BMMs were seeded at a density of 5 × 10^5^ cells per well into six-well plates, cultured overnight in medium without RANKL and starved for more than 6 h. Then, FTY720 was used to pretreat the cells for 2 h, and proteins were extracted after 0, 5, 10, 20, 30, and 60 min of stimulation with RANKL.

The protein samples were separated by 10% sodium dodecyl sulfate-polyacrylamide gel electrophoresis (SDS–PAGE), and the separated proteins were subsequently transferred to nitrocellulose membranes and blocked with 5% BSA for 2 h. The nitrocellulose membranes were incubated overnight at 4°C with the respective primary antibodies, which included the following: anti-c-Fos (Affinity, rabbit, IgG, polyclonal antibody, AF0132, 1 *μ*g/mL), anti-NFATc1 (Santa Cruz, mouse, IgG_1_, 7A6, sc7294, 0.5 *μ*g/mL), anti-integrin B3 (Santa Cruz, mouse, IgG2b, D11, sc365679, 0.5 *μ*g/mL), anti-HDAC4 (Abcam, mouse, IgG, HDAC4-144, ab12171, 1 : 1000), anti-ATF4 (Abcam, rabbit, IgG, polyclonal antibody, ab216839, 2 *μ*g/mL), anti-MEF2C (Abcam, rabbit, IgG, EPR19089-202, ab211493, 0.57 *μ*g/mL), anti-I*κ*Ba (Abcam, rabbit, IgG, E130, ab32518, 0.091 *μ*g/mL), anti-NF-*κ*B p65 (Affinity, mouse, IgG1, BF0382, 1 *μ*g/mL), p-NF-*κ*B p65 (Affinity, rabbit, IgG, polyclonal antibody, AF2006, 1 *μ*g/mL), and B-actin (abclonal, rabbit, IgG, 60, AC026, 1 : 5000). Next, the membrane was incubated with anti-mouse (Immunology, goat, IgG, RS0001, 0.8 *μ*g/mL) or anti-rabbit (Immunology, goat, IgG, RS0002, 0.8 *μ*g/mL) secondary antibody at room temperature for 1 hour. The immunoreactive proteins were detected with ECL.

### 2.6. LPS-Induced Cranial Osteolysis Model and Micro-CT Analysis

A total of 24 C57BL/6 male mice at 8 weeks were randomly divided into 4 groups of 6 mice each: the control group (PBS), LPS (MCE, China) group, and LPS groups treated with low (218 nM) or high (512 nM) concentrations of FTY720 [32–34]. In detail, the mice received LPS (2 mg/ml) and/or FTY720 (218 nM or 512 nM) by local cranial injections (100 *μ* L/session, twice a week for two weeks), and local administration was performed through a gelatin sponge placed in the middle of the skull [35]. Except for the group treated with LPS+218 nM FTY720 in which one experimental animal died; all mice in the other groups survived until sampling. The skulls were removed and fixed with 4% paraformaldehyde for 48 h. Microcomputed tomography (micro-CT) scanning was performed using a high-resolution micro-CT (VivaCT80, SCANCO Medical AG, Switzerland) with X-ray energy settings of 70 kVp and 114 *μ*A. First, the scanning area was selected on the basis of the X-ray image displayed by micro-CT to obtain the tomography image, and then the region of interest was selected and used to construct the 3D graphics and obtain bone-related data [36, 37]. We used micro-CT software to perform three-dimensional reconstruction, and then the reconstructed 3D images were qualitatively and quantitatively analyzed. The median region of the skull and the cranial suture was selected during quantitative analysis, and the indicators included the trabecular number (Tb.N), bone volume/tissue volume (BV/TV), trabecular thickness (Tb.Th), and trabecular separation (Tb.Sp). For micro-CT data collection and analysis, 6 samples were collected from the PBS and LPS groups, and 5 samples were collected from the LPS+FTY720 group (one sample was damaged during skull sampling of the LPS+FTY720 group).

### 2.7. Histomorphometric Analysis

The mouse skulls were fixed with 4% paraformaldehyde and decalcified with 10% EDTA for two weeks, and the decalcifying solution was changed once every two days. Subsequently, gradient dehydration and paraffin embedding were performed, and 5 *μ*m sections were stained with hematoxylin-eosin (H&E) [38, 39] and TRAP. The 5 *μ*m paraffin sections were used for H&E staining by staining with hematoxylin for 3 min and eosin for 1 min. Images were collected after the sections were sealed. Image acquisition was performed using scanning equipment (CaseViewer 2.1, 3DHISTECH Ltd.).

### 2.8. Animal Experiments

The mice used in this study were purchased from the Laboratory Animal Center of Shanxi Medical University (Shanxi, China). All animal protocols were approved by the Institutional Animal Care and Use Committee of the Second Hospital of Shanxi Medical University. The animal use license number is SYXK (jin) 2021-0001. The animals used in the experiment were C57BL/6 mice at 6 or 8 weeks old, and the animals were maintained on a 12-hour day-night cycle with free access to food and water. The C57BL/6 mice were euthanized by CO_2_ intoxication and then immersed in 75% alcohol to extract bone marrow cells for BMM induction or anesthetized to prepare for generation of the skull LPS-induced osteolysis model.

### 2.9. Statistical Analysis

All experiments in this study were independently repeated at least three times. For data analysis, one-way ANOVA with Tukey's post hoc test or two-way ANOVA with Bonserroni's post hoc test was performed based on the data distribution using GraphPad Prism 5 (GraphPad Software). Data are presented as the mean ± standard error of the mean (SEM). Differences for which *P* < 0.05 were considered statistically significant.

## 3. Results

### 3.1. FTY720 Inhibited Osteoclastogenesis in a Dose-Dependent Manner

**Figure 1:**
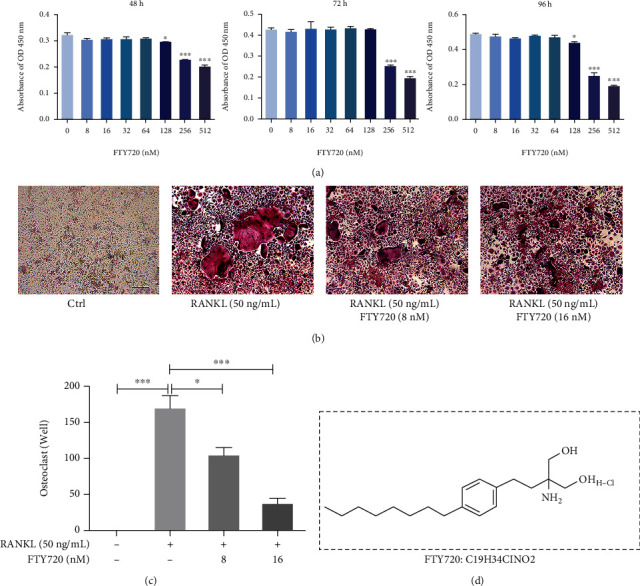
FTY720 inhibits receptor activator of NF-*κ*B ligand- (RANKL-) induced osteoclastogenesis without cytotoxicity. (a) The cell counting kit-8 (CCK-8) assay was used to detect the OD (optical density) value reflecting the level of cell proliferation under treatment with FTY720 for different times (48 h, 72 h, and 96 h) and at different concentrations (8-512 nM) (*n* = 3). (b) Tartrate-resistant acid phosphatase (TRAP) staining of osteoclasts (OCs) in the presence or absence of FTY720. Scale bar: 200 *μ*m. (c) Quantitative analysis of TRAP-positive OCs with three or more nuclei (*n* = 3). (d) The molecular structure of FTY720. Data are expressed as the mean ± SEM. ^∗^*P* < 0.05, ^∗∗^*P* < 0.01, ^∗∗∗^*P* < 0.001 by one-way ANOVA with Tukey's post hoc test (a, c).

To determine the role of FTY720 in OC formation, we first determined the cytotoxicity of FTY720 at a range of concentrations in BMMs by CCK-8 assay. This experiment (Figure 1(a)) confirmed that 0-64 nM FTY720 did not affect the proliferation or survival of BMMs, which allowed us to use FTY720 at this concentration for follow-up experiments. BMMs under OC-induction conditions were treated with FTY720 at different concentrations (0, 8 nM, 16 nM, 32 nM, 64 nM, 128 nM, 256 nM, and 512 nM). As shown in Figures 1(b) and 1(c), osteoclastogenesis was observed by TRAP staining, and multinucleated mature OCs were present in the positive control wells, while the number of OCs in the FTY720-treated group decreased with increasing FTY720 concentration. In addition, the TRAP staining results indicated that FTY720 could inhibit the differentiation and maturation of BMMs into OCs. FTY720 at concentrations of 16 nM and above significantly reduced the number of mature OCs that formed. Based on the CCK-8 assay results, this decrease in OC number was not due to the toxicity of FTY720 but rather to its direct inhibitory effect on OC production and maturation. Therefore, we chose 16 nM FTY720 for subsequent experimental exploration. These results showed that FTY720 had a significant, dose-dependent inhibitory effect on osteoclastogenesis.

### 3.2. FTY720 Downregulated the Expression of Genes Associated with RANKL-Induced Osteoclastogenesis

**Figure 2:**
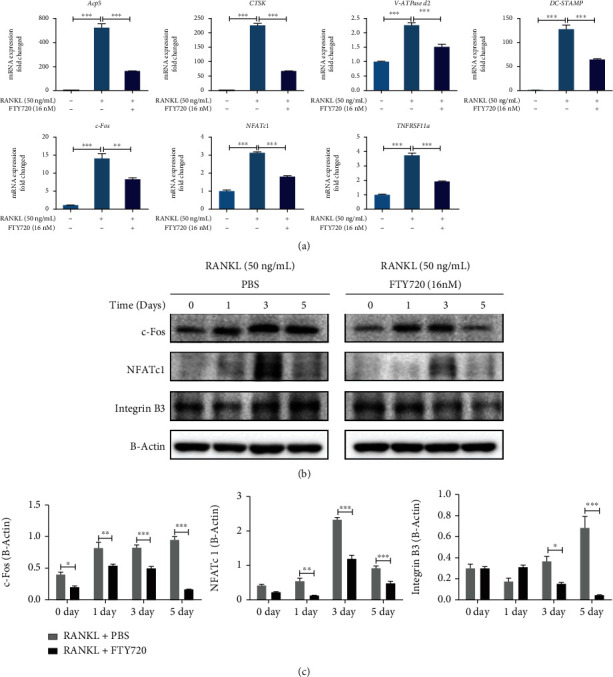
FTY720 downregulates the expression of osteoclast- (OC-) related genes. (a) RT–qPCR detection of the mRNA expression of OC-related genes, including tartrate-resistant acid phosphatase 5 (Acp5), recombinant human cathepsin K (CTSK), vacuolar ATPase d2 (V-ATPase d2), dendritic cell-specific transmembrane protein (DC-STAMP), c-Fos, nuclear factor of activated T cells c1 (NFATc1), and tumor necrosis factor receptor superfamily member 11a (TNFRSF11a). 18 s was selected as the control gene for the experiments (*n* = 3). (b) Western blotting of RANKL- (50 ng/mL) induced expression of c-Fos, NFATc1, and Integrin B3 on days 0, 1, 3, and 5 in the presence or absence of FTY720 (16 nM). B-Actin was selected as the control protein. (c) Quantitative statistics of the grayscale values of the c-Fos, NFATc1, and Integrin B3 protein bands (*n* = 3). Data are expressed as the mean ± SEM. ^∗^*P* < 0.05, ^∗∗^*P* < 0.01, ^∗∗∗^*P* < 0.001 by one-way ANOVA with Tukey's post hoc test (a) or two-way ANOVA with Bonserroni's post hoc test (c).

To confirm that FTY720 inhibits RANKL-induced osteoclastogenesis, we determined the expression levels of OC-specific genes, including Acp5, NFATc1, TNFRSF11a, c-Fos, CTSK, DC-STAMP, and V-ATPase d2. The expression levels of the OC-related genes were significantly higher in the RANKL-treated group than in the control group, but FTY720 downregulated the expression levels of all the genes involved in this process (Figure 2(a)). These genes are involved in the differentiation of BMMs to OCs and OC activity and function and include genes related to fusion (V-ATPase d2 and DC-STAMP), formation (NTATc1 and c-Fos), and even function (Acp5 and CTSK). The expression level of the TNFRSF11a genes, which encodes the RANK proteins, was also reduced by treatment with FTY720 at 16 nM.

To further determine the effect of FTY720 on OCs, the expression levels of several key proteins were detected. Specifically, the expression levels of the key transcription factor NFATc1 and its upstream and downstream proteins during OC differentiation were examined by Western blotting. As shown in Figures 2(b) and 2(c), the expression levels of NFATc1 and c-Fos were significantly increased in RANKL-treated cells after 3 and 5 days of treatment, but FTY720 treatment decreased the expression levels of these two proteins at the same time points. Similarly, FTY720 treatment decreased expression levels of the NFATc1 downstream target protein integrin B3 compared to those in the RANKL-treated group. Both the Western blotting and RT–qPCR results suggested that FTY720 significantly inhibited the expression of genes and proteins related to OC formation and differentiation, further suggesting that FTY720 inhibited osteoclastogenesis and OC function in vitro.

### 3.3. FTY720 Inhibited the NF-*κ*B Signaling Pathway and Upregulated HDAC4 Expression Levels

**Figure 3:**
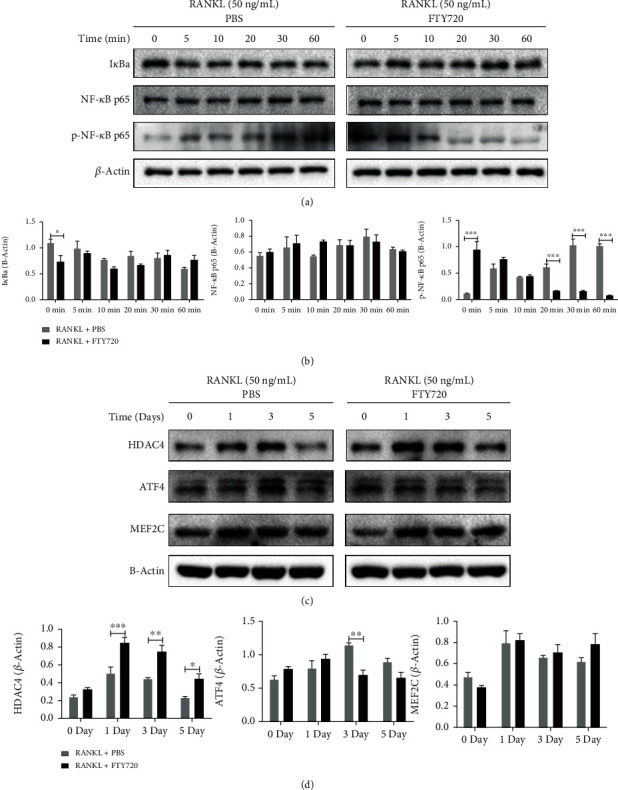
FTY720 suppresses the nuclear factor kappa-B (NF-*κ*B) signaling pathway and upregulates histone deacetylase 4 (HDAC4) expression. (a) Western blotting of I*κ*B*α*, NF-*κ*B p65, and p-NF-*κ*B p65 expression at 0, 5, 10,20, 30, and 60 min under RANKL (50 ng/mL) stimulation with or without FTY720 treatment. B-Actin was selected as the control protein. (b) Quantitative statistics of I*κ*Ba, NF-*κ*B p65, and p-NF-*κ*B p65 expression (*n* = 3). (c) Western blotting analysis of RANKL- (50 ng/mL) induced expression of HDAC4, activating transcription factor 4 (ATF4), and myocyte enhancer factor 2C (MEF2C) on days 0, 1, 3, and 5 in the presence or absence of FTY720. B-Actin was selected as the control protein. (d) Quantitative statistics of HDAC4, ATF4, and MEF2C expression (*n* = 3). Data are expressed as the mean ± SEM. ^∗^*P* < 0.05, ^∗∗^*P* < 0.01, ^∗∗∗^*P* < 0.001 by two-way ANOVA with Bonferroni's post hoc test (b, d).

NF-*κ*B signaling pathways contribute to osteoclastogenesis and OC-specific gene expression [40]. Thus, we further elucidated the detailed inhibitory mechanism by which FTY720 inhibits RANKL-induced osteoclastogenesis. We tested whether FTY720 affects this key pathway in OCs. As expected, RANKL stimulation activated NF-*κ*B p65 phosphorylation and supported OC differentiation. However, pretreatment with FTY720 decreased the RANKL-induced phosphorylation of NF-*κ*B p65. I*κ*Ba and NF-*κ*B p65, the upstream and nonactivated forms of phosphorylated NF-*κ*B p65, respectively, showed no difference in expression levels before and after FTY720 treatment. These results (Figures 3(a) and 3(b)) indicated that FTY720 inhibits OC differentiation by suppressing NF-*κ*B p65 phosphorylation.

As shown in Figures 3(c) and 3(d), the HDAC4 protein level was increased on days 1 and 3 of RANKL-induced osteoclastogenesis and decreased on day 5. HDAC4 protein expression levels in the FTY720-treated group continued to increase on days 1 and 3 and were decreased at day 5, but the HDAC4 expression level in the FTY720-treated group was significantly higher than that in the untreated group. Then, we observed that ATF4 protein levels were significantly increased under RANKL induction for 3 days, while FTY720 treatment downregulated ATF4 expression levels during OC differentiation. Unfortunately, no significant difference in MEF2C protein expression was observed between FTY720-treated cells and controls. Taken together, these data suggest that FTY720 can also promote HDAC4 expression and suppress ATF4 expression, ultimately inhibiting OC differentiation and function.

### 3.4. FTY720 Partially Rescued LPS-Induced Cranial Osteolysis

**Figure 4:**
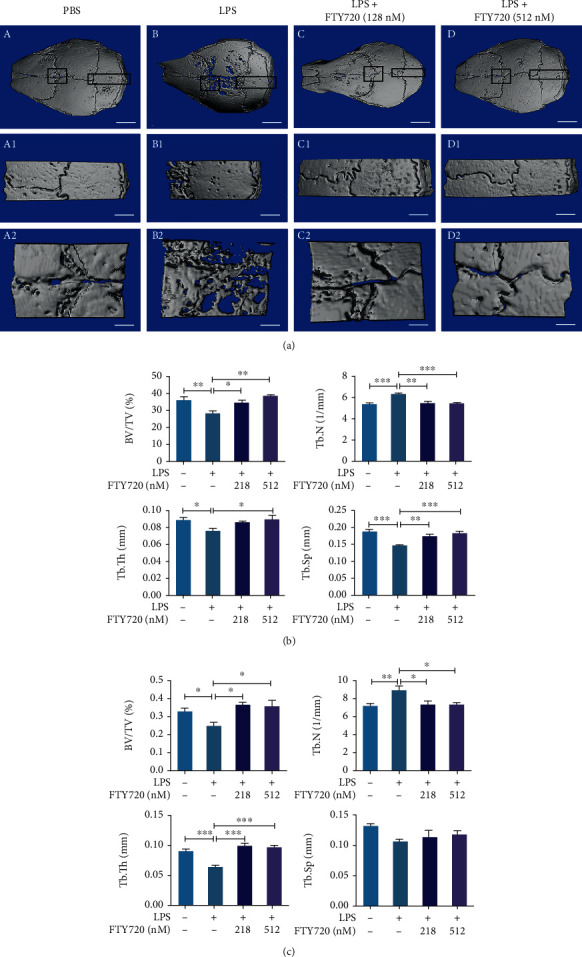
FTY720 restored lipopolysaccharide- (LPS-) induced cranial osteolysis. (a) Reconstruction of cranial bone models by micro-CT upon treatment with PBS, LPS, or LPS and FTY720 (128 nM or 512 nM) together under different conditions. (a–d) 3D reconstructed images of the whole skull with a scale of 2 mm. ((a), A1–D1, A2–D2) The selected region of interest in the median part of the skull and the cranial suture images, respectively, corresponding to scales of 1 mm and 500 *μ*m. (b) Quantitative analysis of the data from the PBS (*n* = 6), LPS (*n* = 6), and LPS with low (*n* = 5) or high (*n* = 5) FTY720 groups collected by micro-CT in the median cranial region for bone volume fraction (BV/TV), trabecular number (Tb.N), subchondral trabecular thickness (Tb.Th), and trabecular separation (Tb.Sp). (c) Quantitative analysis of the data from the PBS (*n* = 6), LPS (*n* = 6), and LPS with low (*n* = 5) or high (*n* = 5) FTY720 groups collected by micro-CT analysis of the cranial suture for BV/TV, Tb.N, Tb.Th, and Tb.Sp. Data are expressed as the mean ± SEM. ^∗^*P* < 0.05, ^∗∗^*P* < 0.01, ^∗∗∗^*P* < 0.001 by one-way ANOVA with Tukey's post hoc test (b, c).

We explored the effect of FTY720 treatment on osteolysis in vivo by establishing a model of LPS-induced cranial osteolysis. The micro-CT results (Figure 4(a)) showed extensive osteolysis on the skull surface in the LPS group; however, treatment with FTY720 at both low and high concentrations significantly attenuated osteolysis. Both of our selected regions of interest, the median skull (Figure 4(a), A1–D1) and the cranial suture (Figure 4(a), A2–D2), showed this effect. In addition, as shown in Figures 4(b) and 4(c), quantitative analysis of bone-related parameters (BV/TV, Tb.N, Tb.Th, and Tb.Sp) confirmed that FTY720 inhibited LPS-induced osteolysis. The BV/TV ratio and Tb.Th and Tb.Sp levels were lower in the LPS group than in the PBS group, but they returned to normal levels in the groups treated with FTY720 at a low or high concentration; however, Tb.N followed the opposite trend. These results indicated that FTY720 could partially restore LPS-induced cranial osteolysis.

### 3.5. FTY720 Inhibited Osteoclastogenesis In Vivo

**Figure 5:**
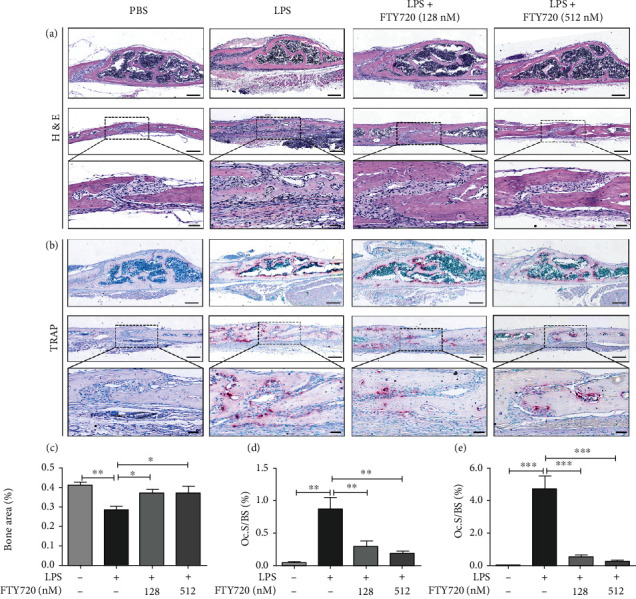
FTY720 treatment partially restored cranial bone morphology and inhibited osteoclastogenesis in vivo. (a) Hematoxylin-eosin (H&E) staining of the region of interest. Median skull, scale bar, 200 *μ*m (top panels), cranial suture, 200 *μ*m (middle panels), and 50 *μ*m (bottom panels). (b) TRAP staining of the region of interest. Median skull, scale bar, 200 *μ*m (top panels), cranial suture, 200 *μ*m (middle panels), and 50 *μ*m (bottom panels). (c–e) Quantitative analysis of the skull middle bone marrow cavity area (c) and the number of TRAP-positive stained osteoclasts in the skull median (d) and on the skull cranial sutures (e) in the PBS (*n* = 6), LPS (*n* = 6), and LPS with low (*n* = 5) or high (*n* = 5) FTY720 groups. Data are expressed as the mean ± SEM. ^∗^*P* < 0.05, ^∗∗^*P* < 0.01, ^∗∗∗^*P* < 0.001 by one-way ANOVA with Tukey's post hoc test (b–d).

To further confirm the protective effect of FTY720 on osteolysis, all cranial bones were histologically evaluated after decalcification. The H&E staining results (Figures 5(a) and 5(c)) showed that the LPS group had a lower bone volume and lower trabecular density in the middle of the skull than the control group, while FTY720 treatment significantly improved the quality of bone at the plate barrier. In addition, the cranial suture was significantly wider and more fibrous in the LPS group, but the cranial bone morphology was partially restored to normal due to the effect of FTY720, and this effect was dose dependent. Although micro-CT did not show a statistically significant difference between the high-dose and low-dose FTY720 groups, the histological results clearly showed that the skull morphology in the high-dose FTY720 group tended to be more similar to that of the controls than that of the low-dose FTY720 group did.

We wanted to determine whether FTY720 would have an effect on OCs in vivo, so we evaluated OCs in skull sections with the assistance of TRAP staining (Figure 5(b)). As shown in Figures 5(b), 5(d), and 5(e), the number of OCs was greater in the LPS group than in the PBS group, and FTY720 treatment reduced the level of positive TRAP staining; with this, effect was more pronounced when FTY720 was applied at high concentrations. This result indicated that FT720 inhibited OC formation and had a protective effect against osteolysis caused by OCs in vivo.

## 4. Discussion

OCs are receiving increasing attention as a major predisposing factor for diseases involving inflammatory bone loss. The nervous system [41] and protein kinase-like endoplasmic reticulum kinase [42] are also involved in osteoclastogenesis and OC function, suggesting that skeletal diseases caused by OC abnormalities are complex. While several articles have reported the potential use of targeting OCs for the treatment of skeletal disorders, drugs that have been clinically used to treat other diseases serve as safer and more reliable options. FTY720 has been used for the clinical treatment of multiple sclerosis, and various studies have shown its potential for the treatment of bone loss disorders. The current findings, which focus on FTY720 as a regulator of S1P, suggest that it also affects osteoclastogenesis [43, 44]. FTY720 had an inhibitory effect on OC formation in ligation-induced periodontitis in rats [45], and study of the role of FTY720 in animal models may provide new ideas for the treatment of periodontitis, which affects 11% of the global population and seriously affects human quality of life [46]. The study of the role of the FTY720 signaling pathway in osteoclastogenesis and OC function not only provides insight into the molecular mechanisms of OCs in skeletal diseases but also may provide a new therapeutic strategy for clinical treatment. However, the detailed molecular mechanism underlying the effects of FTY720 on OC differentiation and OC function has not been clearly reported. In our study, FTY720 treatment significantly decreased the number and size of TRAP-positive cells compared with those observed upon RANKL stimulation alone in vitro. The reduction in the gene expression of RANK (encoded by TNFRSF11a), an irreplaceable and important regulator of OC differentiation [47], after FTY720 treatment, also demonstrated the inhibitory effect of FTY720 on OCs.

RANKL and RANK bind each other on the surface of OC precursor cells, leading to the successive stimulation of NFATc1 and c-Fos [48]. NFATc1 activation is necessary and beneficial for OC formation and induces the expression of many genes involved in OC differentiation and function [9], and NFATc1 is closely associated with inflammation-related osteoporosis [10]. Here, we detected the expression of the main genetic markers of OCs, Acp5, NFATc1, c-Fos, CTSK, DC-STAMP, and V-ATPase d2 by qPCR and found that the mRNA expression levels of these genes increased significantly after RANKL stimulation and remarkably decreased after FTY720 treatment. These findings suggest that FTY720 affects OC formation and fusion and ultimately inhibits OC function. The goal of basic drug research is to relatively clearly elucidate the mechanism of a drug for its application in clinical treatment. We have demonstrated that the addition of FTY720 inhibits the expression of OC marker genes. The important role of NFATc1 in the regulation of OC differentiation and its related gene expression needs to be reevaluated [5]. Consistent with the gene-level changes, FTY720 treatment decreased the levels of NFATc1 and the upstream protein c-Fos compared to those in the RANKL-treated group. Furthermore, NFATc1 downstream targeting of Integrin B3 protein expression increased upon RANKL stimulation and decreased upon FTY720 treatment, further indicating the inhibitory effect of FTY720 on OCs. Our findings suggest that FTY720 attenuates RANKL-induced osteoclastogenesis.

In the present study, we investigated the effect of FTY720 on RANKL-induced OC differentiation and function, and we further investigated the role of FTY720 in an in vivo LPS-induced cranial osteolysis model. The critical role of OCs in inflammatory bone loss makes them a potential target for drug therapy, and LPS is a potent inducer of the immune system that has been used to promote inflammation [49]. Consistent with the results of in vitro experiments, in an in vivo animal model, mice in the LPS group exhibited significantly increased cranial osteolysis and severe bone resorption by micro-CT, while in the same model, FTY720 treatment had an antiresorption effect. Moreover, histomorphometric analysis of the bone showed that FTY720 partially attenuated LPS-induced inflammatory osteolysis in vivo. In addition, TRAP staining results showed more positive staining in the LPS group and less positive TRAP staining after treatment with low or high concentrations of FTY720. Inflammatory bone loss is a complex process that is affected by multiple cellular and signaling pathways [3, 4], and each potential drug may target different signaling pathways. In this in vivo assay, we found that FTY720 improved LPS-induced skull osteolysis by inhibiting OCs. Targeting this mechanism could be one of many solutions to the clinical treatment of these diseases.

The important role of HDAC4 in bone development has been elucidated in several papers, and HDAC4 affects both the proliferation and differentiation of growth plate chondrocytes [50] and regulates anabolism and catabolism during bone development [51]. HDAC4 can promote RANKL expression in osteoblasts [52]. This suggests the importance of exploring the role of HDAC4 in the inhibition of osteoclastogenesis by FTY720. By assessing HDAC4 protein expression, we found that the FTY720-treated group had higher expression levels of HDAC4 than the RANKL-treated group. In contrast, FTY720 treatment decreased RANKL-stimulated ATF4 protein expression levels. Nicholas et al. [14] reported that class IIa HDACs (HDAC4, 5, and 9), which are negative regulators of gene expression, can inhibit OC differentiation. HDAC5 overexpression inhibited RANKL-induced OC differentiation by downregulating the transcriptional activity of NFATc1 [12]. HDAC7 overexpression inhibited osteoclastogenesis through the downregulation of B-catenin [13], and HDAC9 inhibited osteoclastogenesis and bone resorption through a negative regulatory loop with RRAR signaling [53]. Nevertheless, the mechanism by which HDAC4 inhibits OCs has not been reported, and the finding that HDAC4 promotes RANKL expression in osteoblasts [52] strengthens the importance of HDAC4 in OCs. The protein expression of HDAC4 and ATF4 is consistent with changes in endoplasmic reticulum stress-induced apoptosis [15, 16], suggesting that HDAC4 inhibits ATF4, which may also be present in OCs. As early as 2010, Cao et al. reported that ATF4 is a transcription factor in OCs and a direct upstream activator of NFATc1 [18], supporting our results. In addition, MEF2C is involved in multiple HDAC4 responses in cells; more importantly, MEF2C can regulate osteoclastogenesis and pathological bone resorption [54]. However, in RANKL-induced osteoclastogenesis, the protein expression level of HDAC4 was not sufficient to resist the effects of ATF4 and MEF2C in promoting osteoclastogenesis and differentiation. MEF2C protein expression was not altered in any way by FTY720 treatment, indicating that neither FTY720 nor HDAC4 affects OCs via MEF2C. The above results suggest that FTY720 inhibits OC differentiation and function by acting on HDAC4 to suppress ATF4 expression rather than by affecting MEF2C.

Since OCs alter the intracellular microenvironment to affect cancer cell growth, NF-*κ*B signaling is not only a potential target for cancer therapy but also a valuable pathway due to its role in OCs [55]. We also know that RANKL-induced activation of the NF-*κ*B signaling pathway is an important process for OC generation, differentiation, and function [40]. In addition, FTY720 acts as a chemical inhibitor of the Mg ion channel transient receptor potential melastatin 7 (TRPM7) and downregulates NF-*κ*B signaling to affect osteoclastogenesis [56]. In the present study, we found that the phosphorylation level of NF-*κ*B p65 was indeed altered, and a decrease in the expression level of p-NF-*κ*B p65 was observed after 20 min of RANKL stimulation in the presence of FTY720. The activation of p-NF-*κ*B P65 upon RANKL stimulation and the reduction in p-NF-*κ*B P65 levels upon FTY720 treatment could determine whether FTY720 inhibits OC differentiation and function by suppressing activation of the NF-*κ*B signaling pathway. NF-*κ*B is regulated by a variety of cytokines, such as RANKL, IL-1, and TNF- *α*, and occupies a central position in osteolytic diseases such as periprosthetic osteolysis and arthritis, and the use of NF-*κ*B modulators provides new ideas for the treatment of related diseases [57]. Yip et al. showed that parthenolide blocked LPS-induced osteolysis by inhibiting NF-*κ*B activity [58], and in our experiments, FTY720 similarly inhibited NF-*κ*B activity and ultimately affected osteoclastogenesis and LPS-induced skull osteolysis. Here, it seems possible to speculate that FTY720 has capabilities similar to those of other NF-*κ*B modulators, adding new options for designing drugs to treat OC-related diseases.

In this study, both in vivo and in vitro experiments confirmed that FTY720 inhibited RANKL-induced osteoclastogenesis, and we hypothesize that FTY720 inhibits activation of the NF-*κ*B signaling pathway and suppresses ATF4 by upregulating HDAC4 expression levels, which ultimately inhibits OC formation and function. FTY720 partially restored LPS-induced cranial osteolysis in vivo, which also reinforces the importance of exploring the molecular mechanisms by which FTY720 inhibits OC differentiation and function. In addition, a paper suggested that combining FTY720 with biomaterials can significantly promote bone regeneration [59], but the relevant molecular mechanism has not been elucidated, but our experimental results may provide a reference for evaluating possible molecular mechanisms via similar in vivo studies to better advance the study of FTY720 as a potential therapeutic target for the clinical treatment of OC-related diseases. Despite these findings, our study still has some shortcomings. First, HDAC4, as a class IIa HDAC, can shuttle between the nucleus and cytoplasm, and its altered localization in the cytoplasm and nucleus may be important for the expression of targeted genes, but we did not explore this issue in depth in this study. Second, skeletal bone remodeling depends mainly on the balance between bone resorption by OCs and bone formation by osteoblasts, but here, we have not focused on the effect of FTY720 on osteoblasts. More research to explore the molecular mechanism of FTY720 and for its use as a safe and effective clinical drug for the treatment of bone diseases is needed.

## 5. Conclusion

**Figure 6:**
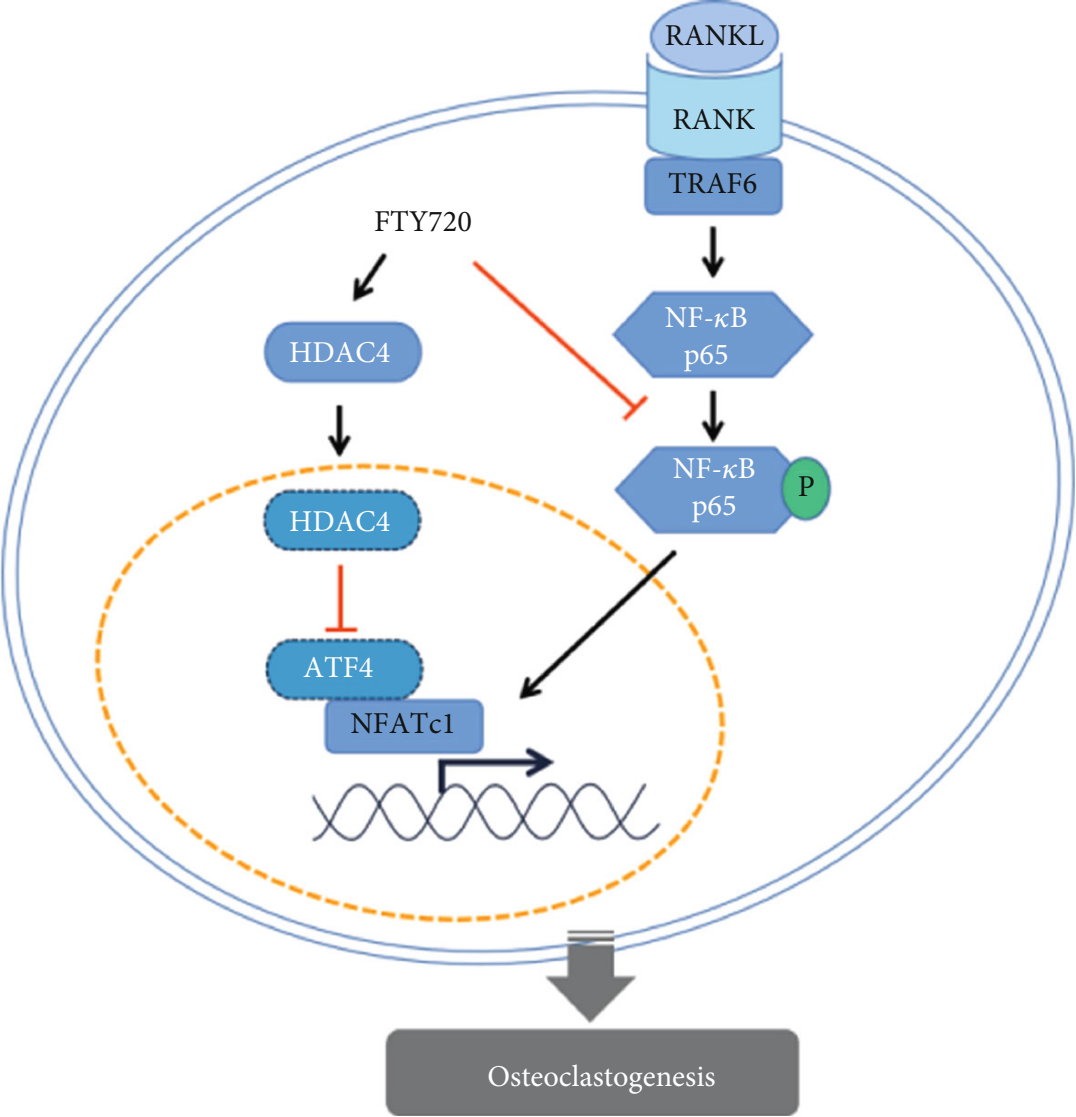
Schematic diagram of the mechanism by which FTY720 inhibits osteoclastogenesis.

FTY720 inhibits RANKL-induced osteoclastogenesis by suppressing the NF-*κ*B signaling pathway and downregulates ATF4 levels by promoting HDAC4 expression (Figure 6). In addition, the protective effect of FTY720 against bone loss was confirmed in vivo using an LPS-induced cranial osteolysis model. The inhibitory effect of FTY720 on OC formation and function renders it, a novel therapeutic target for studying bone and joint diseases due to hyperactivation of OCs.

## Figures and Tables

**Table 1:** Sequences of the primers used for RT-qPCR analysis.

Gene	Froward (5′-3′)	Reverse (5′-3′)
*Acp5*	CAGCAGCCAAGGAGGACTAC	ACATAGCCCACACCGTTCTC
*CTSK*	CCAGTGGGAGCTATGGAAGA	AAGTGGTTCATGGCCAGTTC
*V-ATPase d2*	GTGAGACCTTGGAAGTCCTGAA	GAGAAATGTGCTCAGGGGCT
*DC-STAMP*	TTCATCCAGCATTTGGGAGT	ACAGAAGAGAGCAGGGCAAC
*c-Fos*	TTTCAACGCCGACTACGAGG	GCGCAAAAGTCCTGTGTGTT
*NFATc1*	CAACGCCCTGACCACCGATAG	GGCTGCCTTCCGTCTCATAGT
*TNFRSF11a*	GAAGATGCTTTGGTGGGTGT	TCAGTCGGGATCAGTGTGAG
*18 s*	CGGCTACCACATCCAAGGAA	GCTGGAATTACCGCGGCT

## Data Availability

The data used to support the findings of this study are included within the article.
